# Point-of-Care Ultrasonography in the Diagnosis of Retinal Detachment, Vitreous Hemorrhage, and Vitreous Detachment in the Emergency Department

**DOI:** 10.1001/jamanetworkopen.2019.2162

**Published:** 2019-04-12

**Authors:** Shadi Lahham, Inna Shniter, Maxwell Thompson, Dana Le, Tushank Chadha, Thomas Mailhot, Tarina Lee Kang, Alan Chiem, Stephanie Tseeng, John C. Fox

**Affiliations:** 1 Department of Emergency Medicine, University of California, Irvine; 2 Department of Emergency Medicine, University of Southern California, Los Angeles County, Los Angeles; 3 Department of Emergency Medicine, UCLA (University of California, Los Angeles) Olive View Medical Center, Los Angeles; 4 Department of Emergency Medicine, Loma Linda University, Loma Linda, California

## Abstract

**Importance:**

Ocular symptoms represent approximately 2% to 3% of all emergency department (ED) visits. These disease processes may progress to permanent vision loss if not diagnosed and treated quickly. Use of ocular point-of-care ultrasonography (POCUS) may be effective for early and accurate detection of ocular disease.

**Objective:**

To perform a large-scale, multicenter study to determine the utility of POCUS for diagnosing retinal detachment, vitreous hemorrhage, and vitreous detachment in the ED.

**Design, Setting, and Participants:**

A prospective diagnostic study was conducted at 2 academic EDs and 2 county hospital EDs from February 3, 2016, to April 30, 2018. Patients who were eligible for inclusion were older than 18 years; were English- or Spanish-speaking; presented to the ED with ocular symptoms with concern for retinal detachment, vitreous hemorrhage, or vitreous detachment; and underwent an ophthalmologic consultation that included POCUS. Patients with ocular trauma or suspicion for globe rupture were excluded. The accuracy of the ultrasonographic diagnosis was compared with the criterion standard of the final diagnosis of an ophthalmologist who was masked to the POCUS findings. Seventy-five unique emergency medicine attending physicians, resident physicians, and physician assistants performed ocular ultrasonography.

**Exposure:**

Point-of-care ultrasonography performed by an emergency medicine attending physician, resident physician, or physician assistant.

**Main Outcomes and Measures:**

Sensitivity and specificity of POCUS in identifying retinal detachment, vitreous hemorrhage, and vitreous detachment in patients presenting to the ED with ocular symptoms.

**Results:**

Two hundred twenty-five patients were enrolled. Of these, the mean age was 51 years (range, 18-91 years) and 135 (60.0%) were men; ophthalmologists diagnosed 47 (20.8%) with retinal detachment, 54 (24.0%) with vitreous hemorrhage, and 34 (15.1%) with vitreous detachment. Point-of-care ultrasonography had an overall sensitivity of 96.9% (95% CI, 80.6%-99.6%) and specificity of 88.1% (95% CI, 81.8%-92.4%) for diagnosis of retinal detachment. For diagnosis of vitreous hemorrhage, the sensitivity of POCUS was 81.9% (95% CI, 63.0%-92.4%) and specificity was 82.3% (95% CI, 75.4%-87.5%). For vitreous detachment, the sensitivity was 42.5% (95% CI, 24.7%-62.4%) and specificity was 96.0% (95% CI, 91.2%-98.2%).

**Conclusions and Relevance:**

These findings suggest that emergency medicine practitioners can use POCUS to accurately identify retinal detachment, vitreous hemorrhage, and vitreous detachment. Point-of-care ultrasonography is not intended to replace the role of the ophthalmologist for definitive diagnosis of these conditions, but it may serve as an adjunct to help emergency medicine practitioners improve care for patients with ocular symptoms.

## Key Points

**Question:**

Can emergency medicine physicians use point-of-care ultrasonography to identify retinal detachment, vitreous hemorrhage, and vitreous detachment?

**Findings:**

This prospective diagnostic study of 225 patients presenting to 4 emergency departments with ocular symptoms found that point-of-care ultrasonography demonstrated overall sensitivity of 96.9% and specificity of 88.1% for the diagnosis of retinal detachment, 81.9% sensitivity and 82.3% specificity for vitreous hemorrhage, and 42.5% sensitivity and 96.0% specificity for vitreous detachment.

**Meaning:**

Point-of-care ultrasonography performed by emergency medicine physicians may be a useful adjunct in the diagnosis of retinal detachment, vitreous hemorrhage, and vitreous detachment.

## Teaser

This diagnostic study evaluates the sensitivity and specificity of point-of-care ultrasonography performed by emergency medical practitioners in identifying retinal detachment, vitreous hemorrhage, and vitreous detachment among patients with ocular symptoms who present to the emergency department.

## Introduction

Ocular symptoms are commonly evaluated in the emergency department (ED) and compose approximately 2% to 3% of all ED visits.^1^ These presentations can be benign or can result in permanent vision loss if not quickly identified, diagnosed, and treated. Three common diagnoses encountered in the ED are retinal detachment (RD), vitreous hemorrhage (VH), and vitreous detachment (VD). Of these 3, RD is considered a true ophthalmologic emergency that requires immediate diagnosis and treatment.^2^ Patients with RD may have sudden, painless, monocular vision loss as well as flashes and floaters in the visual field. Similar to RD, symptoms of VH and VD may include vision loss, blurry vision, and visual floaters. Distinguishing between these 3 conditions is clinically important because patients with VH and VD can often be discharged with close outpatient follow-up, whereas patients with RD may need emergency evaluation by an ophthalmologist.

Currently, patients with ophthalmologic symptoms undergo initial testing that includes visual acuity, direct ophthalmoscopy, slitlamp examination, and tonometry.^3^ However, the criterion standard for the establishment of a diagnosis of ocular diseases such as RD is an ophthalmologic evaluation. The diagnosis of ocular disease by an ophthalmologist may entail procedures such as a dilated ophthalmoscopic examination, optical coherence tomography, or ophthalmic ultrasonography.^4,5^ These procedures are used to evaluate the posterior chamber of the eye and clearly visualize the distinct layers of the retina.

Ultrasonography has been used by ophthalmologists for decades to evaluate ocular symptoms but has gained favor by emergency medicine practitioners.^6^ Previous studies have shown that emergency medicine physicians are able to use ocular point-of-care ultrasonography (POCUS) to identify RD in the ED.^7,8,9,10^ However, these studies had limitations, including small sample size, highly trained sonographers, and large confidence intervals. The largest study thus far is a retrospective study that included 142 patients, 34 of whom were found to have RD.^11^ Another large retrospective study included 115 patients, but only 16 received a diagnosis of RD.^12^ To date, no large-scale, prospective, multicenter trials have been performed, to our knowledge, to evaluate the ability of emergency medicine practitioners to diagnose RD, VH, or VD using POCUS.

Our objective was to perform a large-scale, prospective, multicenter study to determine the accuracy of ocular POCUS in the evaluation of RD, VH, and VD. We compared the emergency medicine practitioners’ POCUS diagnosis with the criterion standard of the attending ophthalmologists’ final diagnosis.

## Methods

### Study Design

This study followed the Standards for Reporting of Diagnostic Accuracy (STARD) reporting guideline. We conducted a multicenter, prospective, observational diagnostic study using a convenience sample of patients between February 3, 2016, and April 30, 2018, who presented to the ED with ocular symptoms for which RD, VH, or VD was suspected and who underwent emergent ophthalmologic consultation. Ocular symptoms included blurry vision, flashers and floaters, and vision loss. Four different EDs were used to collect data and enroll patients. Patient enrollment began at different dates owing to site institutional review board approval. The study was approved by all institutional review boards at each of the participating hospitals. Both written and oral informed consent were obtained from each patient prior to enrollment in the study. The University of California, Irvine, UCLA (University of California, Los Angeles), University of Southern California, and Loma Linda University institutional review boards approved the study for their respective sites.

### Study Setting

Of the 4 sites, 2 were academic EDs and 2 were county hospital EDs with academic emergency medicine attending physicians present. All 4 sites support an emergency medicine residency, ophthalmology residency, and emergency ultrasonography fellowship. The combined annual ED census of all 4 sites is greater than 300 000 patient visits per year with a culturally and economically diverse patient population. Twenty-four–hour ophthalmologic consultation was available at all 4 sites.

Seventy-five unique practitioners evaluated patients with ocular symptoms in the ED, including emergency medicine attending physicians, resident physicians, and supervised physician assistants. These practitioners had variable POCUS experience and training. Each site provided annual POCUS training and independent credentialing for all practitioners. Before enrollment, we gave all practitioners a 30-minute lecture followed by 30 minutes of hands-on scanning of healthy volunteer models. The training introduced the practitioner to ocular POCUS and outlined the key sonographic features that distinguish RD, VH, and VD.

### Selection of Participants

Any patient was eligible for enrollment in the study who presented to the ED with ocular symptoms; with a concern for RD, VH, or VD; and undergoing an ED ophthalmologic consultation. Undergraduate research assistants present throughout the various EDs between 8 am and midnight monitored the ED tracking board for eligible patients. Practitioners were approached and asked if the patient had concern for RD, VH, or VD. Patients who met the study criteria and were undergoing an ophthalmologic consultation were approached for enrollment in the study by the research team. We excluded persons younger than 18 years, non-English or non-Spanish speakers, those who declined to be enrolled in the study, and those with ocular trauma or suspicion for globe rupture.

### Study Protocol

All enrolled patients underwent a POCUS performed by the treating practitioner. To ensure that the practitioners were not influenced by the ophthalmologic examination results, POCUS was performed before the patient’s ophthalmologic consultation. The ophthalmologist who examined the patient was masked to the results of the POCUS. Ocular POCUS was performed using the following ultrasound machines: Mindray TE7 (Mindray North America) and Sonosite M-Turbo (FUJIFILM Sonosite). All POCUS machines were equipped with a linear, high-frequency probe at 7.5 MHz with a dedicated ophthalmologic setting. This setting produced a thermal index less than 1.0 and a mechanical index less than 0.23.

Patients were placed in an upright or supine position based on practitioner preference. Ultrasound gel was applied to the upper eyelid and the linear ultrasound transducer was placed over the patient’s closed eyelid. Both sagittal and transverse views of the affected eye were obtained. In the transverse orientation, the probe marker was aimed to the patient’s right; in the sagittal orientation, it was aimed cephalad. With the use of ultrasonography, the posterior chamber of the globe was inspected for the presence of an RD, VH, or VD. Depth and gain were set at the discretion of the treating practitioner. Practitioners performed both static and kinetic examinations to aid in distinguishing among the 3 conditions. During a static examination, the patient held the eye still and the sonographer fanned through the globe. During a kinetic examination, the sonographer held the probe steady and the patient was instructed to look left and right.

**Figure 1.**
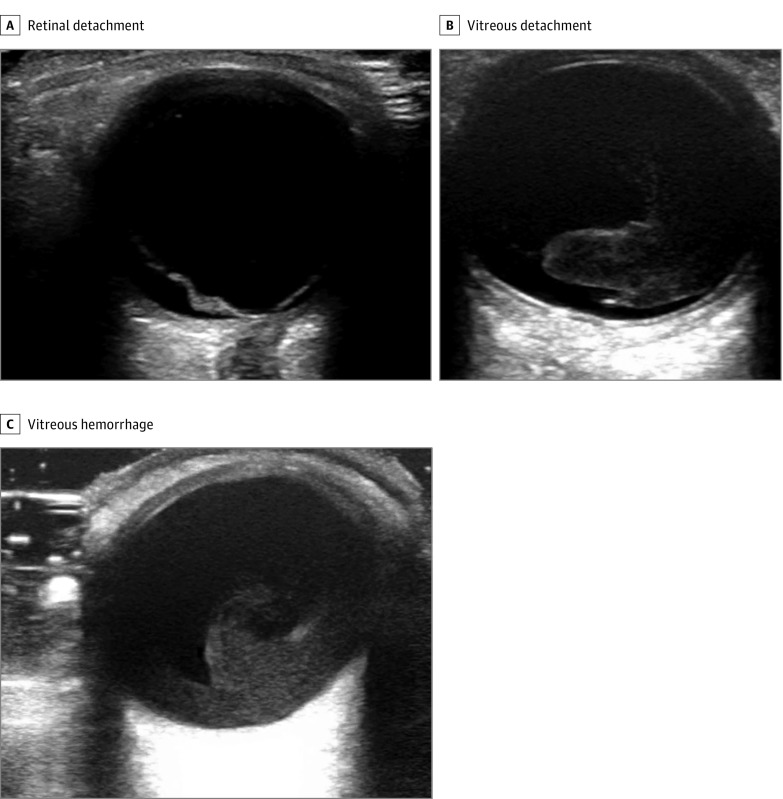
Point-of-Care Ultrasonographic Images A, Retinal detachment. A bright, echogenic membrane is tethered to the optic disc but separated from the choroid in the far field of the image. B, Vitreous detachment. A detached, thin, mobile membrane can be seen at the interface between the vitreous and the retina. C, Vitreous hemorrhage. A fluid collection of variable echogenicity can be seen in the posterior chamber.

The entire orbit was scanned by the practitioner in a fanning motion. B-mode ultrasonography was used to visualize the patient’s vitreous body and posterior chamber. An RD was confirmed by the presence of a bright, echogenic membrane tethered to the optic disc but separated from the choroid (Figure 1A). A posterior VD was defined by the presence of a detached, thin, mobile membrane at the interface between the vitreous and the retina (Figure 1B). These 2 abnormalities were differentiated based on the visual appearance of the membrane and whether the membrane was tethered to the optic nerve. A VH was defined by the presence of a fluid collection of variable echogenicity in the posterior chamber that rotated with kinetic examination (Figure 1C). These findings were recorded immediately on a standardized data collection sheet by research personnel at bedside following POCUS. The ultrasonographic diagnoses of the emergency medicine practitioners were compared with the criterion standard of the ophthalmologists’ final diagnoses after their evaluation. For several patients, more than 1 diagnosis was recorded.

### Statistical Analysis

Data were collected by research assistants using portable electronic devices at bedside and transferred to a spreadsheet (Microsoft Excel 2016, 32-Bit Edition; Microsoft Corp). Data were analyzed using Stata, version 10 (StataCorp). The primary end point of the study was the diagnostic accuracy of ocular POCUS in the evaluation of RD, VH, and VD. We calculated the sensitivity, specificity, positive predictive value, negative predictive value, and accuracy with 95% CIs for ocular POCUS compared with the ophthalmologist’s final diagnosis for RD, VH, and VD. These measures are calculated in the standard manner with 95% CIs and continuity correction. The clustered structure of the study sample on physicians’ level was taken into account while calculating the 95% CI of the sensitivity, specificity, and predictive values. For this, we used the XTGEE command in Stata/SE, version 14.2 (StataCorp) and the robust vce option. For a combination of RD, VH, or VD, we expected that ultrasonography would be at least 80% sensitive based on previous data. Thus, we calculated a sample size of 225 patients using an estimated 15% incidence of RD, VH, or VD in our population. Statistical significance was also calculated in Stata, version 10 using a 2-tailed test. Significance was determined as *P* < .05.

## Results

We approached 252 patients for enrollment in the study and excluded 27 patients from the final data analysis for the following reasons: 13 patients declined to be enrolled, 8 patients had incomplete data collection, 4 patients did not receive ophthalmologic consultation in the ED, and 2 patients requested to be removed from the study following enrollment. Two hundred twenty-five patients were included in the final data analysis. One hundred thirty-five (60.0%) of the patients were men and 90 (40.0%) were women. The mean (range) age was 51 (18-91) years. Chief concerns included blurry vision, vision loss, and flashers and floaters. Seventy-five unique practitioners were used to enroll patients, including 70 emergency medicine physicians and 5 physician assistants. A minimum number of enrolled patients per practitioner was 1 and a maximum number of enrolled patients per practitioner was 8, with a median of 3.6. Of the emergency medicine physicians, 20 were attending physicians, 8 were postgraduate year (PGY)–4, 17 were PGY-3, 11 were PGY-2 and 14 were PGY-1. Of the 225 patients, 173 were included in data analysis from the University of California, Irvine Medical Center ED; 34 patients were included from the Los Angeles County + University of Southern California ED; 14 patients were included from the UCLA Olive View Medical Center ED; and 4 patients were included from the Loma Linda University Medical Center ED.

**Figure 2.**
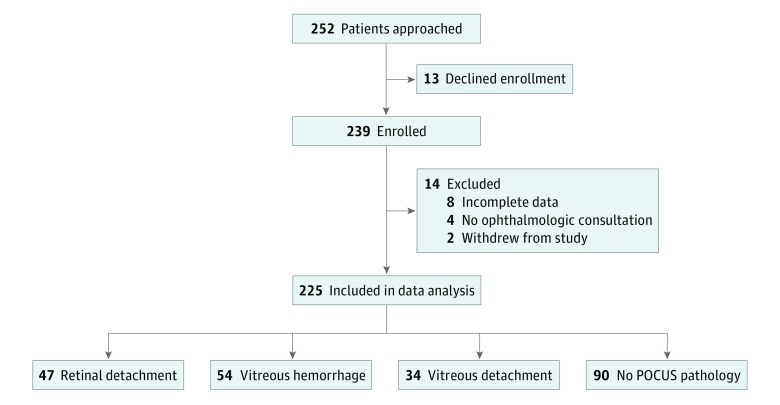
Flow Diagram Illustrating the Number of Patients Enrolled and Excluded and the Various Categories for Each Patient Group POCUS indicates point-of-care ultrasonography.

Of the 225 patients included in data analysis, 47 (20.8%) received a diagnosis of RD; 54 (24.0%), VH; and 34 (15.1%), VD by an ophthalmologist (Figure 2). The prevalence of disease was 36%. Emergency department–performed ocular POCUS correctly identified RD in 46 of the 47 confirmed cases, resulting in an overall sensitivity of 96.9% (95% CI, 80.6%-99.6%). Point-of-care ultrasonography accurately ruled out 156 of 176 cases determined by ophthalmologists to be negative for RD, resulting in a specificity of 88.1% (95% CI, 81.8%-92.4%). Ocular POCUS was able to identify 46 of 54 cases of VH, resulting in an overall sensitivity of 81.9% (95% CI, 63.0%-92.4%). The specificity for VH was 82.3% (95% CI, 75.4%-87.5%). In contrast to RD and VH, ocular POCUS correctly identified VD in 19 of 34 patients, resulting in a sensitivity of 42.5% (95% CI, 24.7%-62.4%). However, ocular POCUS was able to accurately rule out 178 of 190 cases determined by an ophthalmologist to be negative for VD, resulting in a specificity of 96.0% (95% CI, 91.2%-98.2%). The pooled sensitivities, specificities, positive predictive values, and negative predictive values for the 3 disease processes are listed in the Table.

**Table.** Diagnostic Factors of Point-of-Care Ultrasonography for Retinal Detachment, Vitreous Hemorrhage, and Vitreous Detachment

Factor	% (95% CI)		
	Retinal Detachment	Vitreous Hemorrhage	Vitreous Detachment
Sensitivity	96.9 (80.6-99.6)	81.9 (63.0-92.4)	42.5 (24.7-62.4)
Specificity	88.1 (81.8-92.4)	82.3 (75.4-87.5)	96.0 (91.2-98.2)
Positive predictive value	64.5 (49.8-76.9)	46.0 (40.4-51.6)	61.3 (37.8-80.4)
Negative predictive value	99.0 (94.0-99.8)	94.3 (86.6-97.7)	91.8 (84.8-95.7)
Accuracy	90.6 (86.0-94.1)	79.9 (74.1-85.0)	88.0 (83.0-91.9)

## Discussion

Ocular POCUS is a diagnostic modality that may aid emergency medicine practitioners in identifying vision-threatening ocular disease processes.^13^ Point-of-care ultrasonography is ideal for the ED setting because of its portability, lack of radiation exposure, and time efficiency. Using POCUS to evaluate ocular pathology is promising because the eye is superficial and fluid filled. The available literature has shown that emergency medicine practitioners can detect ocular anomalies using ocular POCUS.^7,8,9,10,11,14^ Blaivas et al^7^ prospectively enrolled 61 participants to assess the accuracy of POCUS for evaluating general ocular disease processes and found a sensitivity of 100% and specificity of 97.2%. A 2017 study by Baker et al^10^ showed that emergency medicine practitioners are able to differentiate between RD and VD with moderate accuracy; in that study, the sensitivity for RD was 74.6% and the sensitivity for posterior VD was 85.7%.

Thus far, 2 retrospective studies and 3 prospective studies have demonstrated the utility of POCUS specifically in diagnosing RD in urban and suburban academic EDs. A retrospective study of 109 patients conducted by Jacobsen et al^11^ found that ocular POCUS detected RD with a sensitivity of 91% and specificity of 96%. In a prospective study by Yoonessi et al,^8^ 48 patients were enrolled, and POCUS exhibited a sensitivity of 100% and specificity of 83%. Shinar et al^9^ also conducted a prospective study that recruited 90 patients and determined a sensitivity of 97% and specificity of 92%. Most recently, Kim et al^12^ conducted a prospective study enrolling 115 patients, with a sensitivity of 75% and specificity of 94%. This sensitivity was substantially lower than in our study. However, in the study by Kim et al, trainees (residents and fellows) exhibited a sensitivity of 100% and specificity of 95%. This difference may be associated with the increased POCUS training for medical students and residents. To our knowledge, no studies have evaluated POCUS experience with the ability to identify RD, VH, or VD.

To date, this study is the largest prospective study and the first multicenter study, to our knowledge, investigating the utility of POCUS to diagnose ocular disease processes in the ED. A recent systematic review and meta-analysis performed by Gottlieb et al^15^ confirms our findings regarding POCUS for the identification of RD. However, to our knowledge, no other studies have evaluated the sensitivities and specificities of ocular POCUS in the diagnosis of VD and VH in addition to RD. In the study by Gottlieb et al,^15^ 5 studies were performed in the ED and resulted in a sensitivity of 92.0% and a specificity of 91.4%. Our data indicated a higher sensitivity but lower specificity. Several factors can account for these differences, including variable ultrasonography equipment, variability in the training protocol, and sonographer experience. Regardless of these variables, our data support the findings of previous retrospective, prospective, and systematic review studies showing that emergency medicine practitioners can diagnose retinal detachment with high accuracy using POCUS. Because RD may result in irreversible vision loss, the ability to detect it promptly may be useful in improving transition of care from emergency medicine to ophthalmology, substantiating the need for these patients to receive emergency consultation.

Emergency medicine practitioner–performed POCUS was not as sensitive in identifying VD and was only modestly accurate at diagnosing VH. However, the higher specificities for these 2 pathologies indicate that emergency medicine practitioners are better at successfully ruling in these conditions. The lower sensitivities may have been associated with the fact that most emergency medicine practitioners are more focused on finding RD or are more comfortable identifying RD than VD and VH. These 2 disease processes, unlike RD, are not considered true ophthalmologic emergencies, and these patients may be referred to an ophthalmologist for prompt outpatient follow-up. The ability to accurately differentiate between these ocular disease processes may be useful for determining the urgency with which patients would need to be examined by an ophthalmologist.

We believe that, given the results of our data, POCUS can be used by emergency medicine practitioners to quickly identify RD, VH, and VD in the ED. The addition of POCUS to the history and physical examination provides a useful adjunct method to confer additional information to the ophthalmologist.

### Limitations

There are several limitations to this study. Our study was conducted within 4 different EDs. Two of the sites were academic EDs and 2 were county EDs; thus, it is unclear whether the findings will translate to patient populations in different settings. Point-of-care ultrasonography is also operator dependent, and our sonographers had varying levels of ultrasonography experience and proficiency. The amount of training required for proficiency in ocular POCUS was not addressed in this study. Interrater reliability was not evaluated in this study but should be considered in future studies. An additional limitation of our study was convenience sampling because our research team was able to enroll patients daily only from 8 am to midnight despite the availability of 24-hour ophthalmologic services. Although our study primarily evaluated the use of POCUS to diagnose RD, we did not ask our sonographers to specifically distinguish macula-on from macula-off detachments or retinal tears. Diagnoses other than RD, VH, and VD were not evaluated using POCUS and should be considered in patients with ocular symptoms presenting to the ED. Patients with globe rupture and possible traumatic RD were excluded from the study; therefore, our results may not be generalizable to this population. Physicians performing POCUS were not masked to the patient’s history or physical examination results; thus, the independent contribution of POCUS is unknown.

## Conclusions

Our findings suggest that emergency medicine practitioners are capable of accurately identifying and differentiating among RD, VH, and VD. Point-of-care ultrasonography is not intended to replace the role of the ophthalmologist for definitive diagnosis of these conditions; it serves as an adjunct method to help emergency medicine practitioners improve care for patients with ocular symptoms. This diagnosis method may be of particular benefit to EDs where around-the-clock ophthalmologic consultation may not be accessible.
